# Smoking and life expectancy among HIV-infected individuals on antiretroviral therapy in Europe and North America

**DOI:** 10.1097/QAD.0000000000000540

**Published:** 2015-01-07

**Authors:** Marie Helleberg, Margaret T. May, Suzanne M. Ingle, Francois Dabis, Peter Reiss, Gerd Fätkenheuer, Dominique Costagliola, Antonella d’Arminio, Matthias Cavassini, Colette Smith, Amy C. Justice, John Gill, Jonathan A.C. Sterne, Niels Obel

**Affiliations:** a Department of Infectious Diseases, Copenhagen University Hospital, Rigshospitalet; b Faculty of Health Sciences, University of Copenhagen, Copenhagen, Denmark; c School of Social and Community Medicine, University of Bristol, Bristol, UK; d Université Bordeaux, ISPED, Centre INSERM U897-Epidémiologie-Biostatistique, Bordeaux, France; e Department of Global Health, Academisch Medisch Centrum bij de Universiteit van Amsterdam, and Stichting HIV Monitoring, Amsterdam, The Netherlands; f Department of Internal Medicine, University of Cologne and German Centre for Infection Research (DZIF), Cologne, Germany; g Sorbonne Universités, UPMC Univ Paris 06; h INSERM, UMR_S 1136, Institut Pierre Louis d’Epidémiologie et de Santé Publique, Paris, France; i Clinic of Infectious Diseases & Tropical Medicine, San Paolo Hospital, University of Milan, Milan, Italy; j Service des maladies infectieuses, CHUV, Lausanne, Switzerland; k Research Department of Infection and Population Health, University College London, London, UK; l Yale University, New Haven; m VA Connecticut Healthcare System, West Haven, Connecticut, USA; n Division of Infectious Diseases, University of Calgary, Calgary, Alberta, Canada.

**Keywords:** HIV, life expectancy, mortality, non-AIDS related mortality, smoking, tobacco

## Abstract

**Background:**

Cardiovascular disease and non-AIDS malignancies have become major causes of death among HIV-infected individuals. The relative impact of lifestyle and HIV-related factors are debated.

**Methods:**

We estimated associations of smoking with mortality more than 1 year after antiretroviral therapy (ART) initiation among HIV-infected individuals enrolled in European and North American cohorts. IDUs were excluded. Causes of death were assigned using standardized procedures. We used abridged life tables to estimate life expectancies. Life-years lost to HIV were estimated by comparison with the French background population.

**Results:**

Among 17 995 HIV-infected individuals followed for 79 760 person-years, the proportion of smokers was 60%. The mortality rate ratio (MRR) comparing smokers with nonsmokers was 1.94 [95% confidence interval (95% CI) 1.56–2.41]. The MRRs comparing current and previous smokers with never smokers were 1.70 (95% CI 1.23–2.34) and 0.92 (95% CI 0.64–1.34), respectively. Smokers had substantially higher mortality from cardiovascular disease, non-AIDS malignancies than nonsmokers [MRR 6.28 (95% CI 2.19–18.0) and 2.67 (95% CI 1.60–4.46), respectively]. Among 35-year-old HIV-infected men, the loss of life-years associated with smoking and HIV was 7.9 (95% CI 7.1–8.7) and 5.9 (95% CI 4.9–6.9), respectively. The life expectancy of virally suppressed, never-smokers was 43.5 years (95% CI 41.7–45.3), compared with 44.4 years among 35-year-old men in the background population. Excess MRRs/1000 person-years associated with smoking increased from 0.6 (95% CI –1.3 to 2.6) at age 35 to 43.6 (95% CI 37.9–49.3) at age at least 65 years.

**Conclusion:**

Well treated HIV-infected individuals may lose more life years through smoking than through HIV. Excess mortality associated with smoking increases markedly with age. Therefore, increases in smoking-related mortality can be expected as the treated HIV-infected population ages. Interventions for smoking cessation should be prioritized.

## Introduction

To target health interventions where they are most needed, it is necessary to evaluate which risk factors are major contributors to loss of potential life years. The contribution of risk factors to the global burden of disease has shifted from communicable towards noncommunicable diseases, and tobacco smoking was the leading risk factor for disease burden in North America and Western Europe in 2010 [1].

Morbidity and mortality among people living with HIV decreased substantially after the introduction of combination antiretroviral therapy (ART) in 1996 [2,3]. With the decline in AIDS mortality, cardiovascular diseases (CVDs) and non-AIDS malignancies have become major causes of death in the HIV-infected population [4–7]. All-cause mortality as well as the risk of non-AIDS related death continues to be higher among HIV-infected individuals than among the background population [8,9]. The relative contributions of immune deficiency, inflammation, cumulative toxicities of long-term ART and lifestyle to mortality in HIV-infected individuals remain unclear [10,11].

A recent study [12] in Denmark found that more than 60% of deaths in the HIV-infected population were associated with smoking, and that the number of life years lost in association with smoking was larger than that associated with HIV-related factors among 35-year-old HIV-infected smokers. We hypothesized that these results can be generalized to Western Europe and North America and that smoking and lifestyle-associated factors may contribute more to excess mortality in the treated HIV-infected population than factors related to HIV. The aims of the present study were to estimate associations of smoking with all-cause and cause-specific mortality and to estimate loss of life years associated with smoking, among HIV-infected individuals treated in Western Europe and North America. Furthermore, we aimed to assess whether the association between smoking and mortality differ by age or immune status.

## Materials and methods

### Participants

The ART Cohort Collaboration (ART-CC) is an international collaboration between the investigators of cohort studies of HIV-1 positive individuals from Europe and North America, described in detail elsewhere [13] and at www.art-cohort-collaboration.org. Prospective cohort studies were eligible to participate if they had enrolled at least 100 HIV-1 positive individuals aged at least 16 years who had not previously received ART, started ART with a combination of at least three antiretroviral drugs and had a median duration of potential follow-up of at least 1 year after ART initiation. All cohorts provided anonymized data on a predefined set of demographic, laboratory and clinical variables, which were then pooled and analysed centrally. The dataset analysed here included data from eight cohorts, which are listed in the web appendix. The numbers of individuals included in the present study from each cohort are summarized in supplementary table 2. The NHS Health Research Authority South West, Cornwall and Plymouth Research Ethics Committee, UK, approved the study (REC reference 12/SW/0253).

### Participant selection and data collection

Individuals were eligible for inclusion in this analysis if they started ART between 1 January 1996 and 1 December 2008, were alive and under follow-up 365 days after initiation of ART, and data on smoking status were available. Follow-up time started (baseline) at the latest of first date of ascertainment of smoking status or 365 days after ART initiation, by which time CD4^+^ cell counts have usually risen substantially, with corresponding declines in AIDS-related mortality [7,14,15]. Individuals whose transmission was via injection drug use were excluded, because the vast majority are smokers and because they experience high mortality due to comorbidities and other nonsmoking risk factors [16]. Thus, if IDUs were included in analyses, there would be a risk of overestimating the impact of smoking on mortality.

Information on mortality was obtained either through linkage with Vital Statistics agencies and hospitals or through physician report and active follow-up of cohort participants. We adapted the Cause of Death (CoDe) project protocol [17] (www.cphiv.dk/CoDe.aspx) to classify causes of death. We used a computer algorithm developed by the Mortalité 2000–2005 Study Group [18] to classify deaths wherein ICD10 codes were available. These classifications were compared with those coded by a clinician. When ICD10 codes were not available, two clinicians independently classified each death. Disagreements between clinicians and/or computer-assigned codes were resolved via panel discussion, as described previously [7]. Deaths were coded as AIDS-related if there was a serious AIDS-defining condition(s) close to death and/or a low CD4^+^ cell count (<100 cells/μl) prior to death and a diagnosis compatible with AIDS as a cause of death. All other deaths, including deaths of unknown cause, were considered non-AIDS related.

The exposure of interest was tobacco smoking. Individuals were categorized as smokers (current or previous smokers) or nonsmokers on the basis of information on smoking status at baseline and did not change category during the observation period. More detailed data were available on a subgroup of patients from five cohorts, enabling categorization of individuals as current, previous or never smokers.

### Statistical methods

Follow-up time was calculated from baseline until date of death, loss to follow-up, 180 days after last clinical follow-up or 31 December 2009, whichever came first. Excess mortality rates were estimated using the formula: MR_unexposed_ – MR_exposed_. Mortality rate ratios (MRRs) were estimated using Poisson regression adjusted for sex, age (in 2-year intervals), route of HIV transmission, CD4^+^ cell count at baseline, year of ART initiation, years on ART (time-updated in 1-year intervals) and AIDS at ART initiation.

We used abridged life tables to estimate life expectancies among men: there were too few deaths among nonsmoking women to provide meaningful estimates. Abridged life tables describe the mortality that hypothetical cohorts of HIV-infected individuals would have if they were subject to the age-specific mortality rates observed during follow-up. Further details on estimating life expectancy have been described previously [19]. Age-specific mortality rates were calculated for ages 25–65 years in 10-year intervals. Deaths and observation time were pooled for those above 65 years of age. Mortality rates among HIV-infected men were compared with the corresponding mortality rates from the male French general population (www.mortality.org), under the assumption that the French population was representative for Western Europe and North America. In these analyses, the mortality rates of the HIV-infected population were adjusted to the sex-specific smoking frequency in the French population by weighted averages. At ages above 65 years, data were sparse and mortality rates were adjusted, assuming that the MRR of individuals aged at least 65 years compared with those aged 55–65 years was at least 50% of the corresponding MRR in the general French population. In sensitivity analyses, we assumed that the mortality rate of individuals in the HIV population aged at least 65 years was 20% higher and 20% lower than that of the primary analysis. Numbers of life years lost were estimated by subtraction of the age-specific life expectancy of the exposed from that of the unexposed. Stata statistical software, Version 11.0 (StataCorp, College Station, Texas, USA) and Microsoft Excel 2007 were used for data analysis.

### Role of funding sources

The funding sources were not involved in study design, collection, analysis or interpretation of data, in writing of the report or in the decision to submit the article for publication

## Results

### Participants

Of 45 812 eligible HIV-infected individuals in the eight participating cohorts, 3567 were excluded due to IDU and 24 250 were excluded due to lack of data on smoking status, leaving 17 995 individuals in the study who were followed for a total of 79 760 person-years. The proportion of men was 71.3%; 70.6% had viral load less than 400 copies/ml and 56.2% had CD4^+^ cell count more than 350 cells/μl at baseline (Table 1). Compared with nonsmokers, the median age of smokers was 2 years older, the proportion of MSM was higher and duration of ART at baseline was longer. In three of the participating cohorts, individuals were categorized as smokers or nonsmokers (*n* = 9476; 46 777 person-years), and in five of the cohorts, individuals were categorized as current, previous or never smokers (*n* = 8519; 32 983 person-years). Comparing individuals who were excluded with those included in the study, the proportion of men was lower (66.3 versus 71.3%), a higher proportion was aged more than 50 years at ART initiation (17.9 versus 14.1%) and a higher proportion died during follow-up (7.8 versus 2.9%), (supplementary table 1).

### Risk of death among smoking compared with nonsmoking HIV-infected individuals

Rates of all-cause mortality were 7.9 [95% confidence interval (95% CI) 7.2–8.79] per 1000 person-years among smokers and 4.2 (95% CI 3.5–5.0) among nonsmokers; the adjusted MRR comparing smokers with nonsmokers was 1.94 (95% CI 1.56–2.41). MRRs comparing smokers with nonsmokers were similar within strata defined by sex, calendar period, route of HIV transmission, immune status or viral suppression at baseline (Table 2). Among individuals with more detailed smoking data available (*n* = 8519), the proportions of current, previous and never smokers were 46.5, 25.7 and 27.8%, respectively. The MRR among current versus never smokers was 1.70 (95% CI 1.23–2.34) and that of previous versus never smokers was 0.92 (95% CI 0.64–1.34).

### Non-AIDS related deaths

Causes of death could be classified in 452 of 520 cases (90%), of which 152 (29%) were classified as AIDS-related [mortality rate 1.6 per 1000 person-years (95% CI 1.3–1.9)]. The remaining 368 deaths (71%) were considered non-AIDS related [mortality rate 4.6 per 1000 person-years (95% CI 4.2–5.1)]. Rates of non-AIDS related death were increased among smokers compared with nonsmokers [MRR 2.61 (95% CI 1.88–3.61)] (Table 3). Rates of death caused by CVD, non-AIDS malignancy and liver disease were substantially higher among smokers than among nonsmokers [MRR 6.28 (95% CI 2.19–18.0), 3.13 (95% CI 1.80–5.45) and 8.70 (95% CI 1.14–66.6), respectively]. The relative risks of death from CVD, non-AIDS malignancies, infections and liver disease comparing current with never smokers were higher than the relative risks comparing previous with never smokers (Table 3).

### Deaths from non-AIDS malignancies

In supplementary table 3 deaths caused by non-AIDS malignancies are displayed according to cancer site. The proportion of non-AIDS malignant deaths that was caused by lung cancer was 36%. Thirty-four smokers and no nonsmokers died of lung cancer. Of the 94 non-AIDS malignant deaths, 47 (50%) were caused by cancer types strongly related to smoking (lung, head-and-neck, oesophagus, pancreas and bladder cancer) and 45 (96%) of these occurred among smokers.

### Life expectancies and life years lost

Among 35-year-old HIV-infected men, smokers lost 7.9 (95% CI 7.1–8.7) potential life years compared with nonsmokers (Table 4). The life expectancy of 35-year-old HIV-infected men was on average 5.9 (95% CI 4.9–6.9) years shorter than that of 35-year-old men in the background population after adjusting for smoking in the background population. At age 65 years, the loss of life years associated with smoking was 6.6 (95% CI 6.0–7.2) years, whereas that associated with HIV was only 2.9 (95% CI 2.1–3.7) years. Among 35-year-old HIV-infected individuals with CD4^+^ cell count at least 200 cells/μl or viral load less than 400 copies/ml at baseline, the loss of life years associated with HIV was 3.0 (95% CI 2.0–4.0) and 4.0 (95% CI 2.4–5.6) years, respectively, while that associated with smoking was 5.6 (95% CI 4.8–6.4) and 8.5 (95% CI 6.9–10.1) years.

### Excess mortality rates associated with smoking and HIV-related factors

**Fig. 1:**
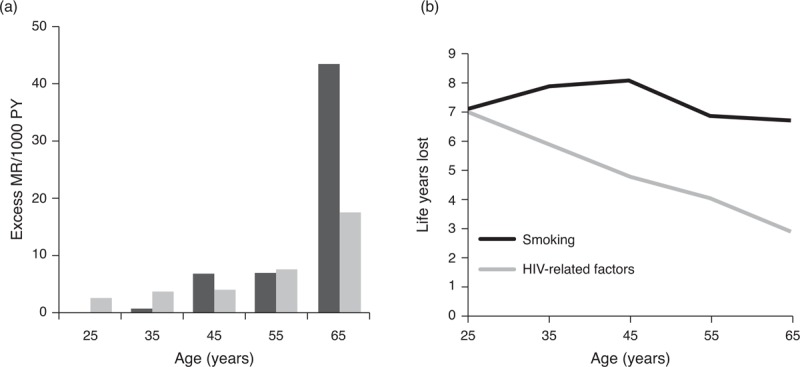
Excess mortality and loss of life years.

Excess mortality rates associated with smoking and HIV-related factors increased with age (Fig. 1). However, the increase in excess mortality rates with age was substantially higher in association with smoking than HIV-related factors. In the younger age groups, the excess mortality rate associated with smoking was limited, but due to the high excess mortality rate at older age, young smokers had a substantially shorter life expectancy than nonsmokers. In the older age groups, HIV-related factors contributed only little to loss of potential life years.

Age-specific excess mortality rates (a) and numbers of life years lost (b) in association with smoking (black bars/line) and HIV-related factors (grey bars/line) among HIV-infected men. PY, person-years.

### Sensitivity analyses

Due to the limited observation time of individuals aged at least 65 years, we examined the robustness of estimates of life expectancies and life years lost in association with smoking in sensitivity analyses wherein the mortality rates of this age group was set 20% lower (model 1) and 20% higher (model 2) than the primary analyses. In these analyses, life expectancies varied by 2–4 years and life years lost in association with smoking varied by 1–2 years compared with the primary analyses (supplementary table 4).

## Discussion

In a large collaborative HIV cohort study including European and North American HIV-infected individuals who had been on ART for at least 1 year, smoking was associated with a two-fold increase in mortality. Deaths from non-AIDS related malignancies and CVD accounted for most of the excess mortality among smokers. More than one-third of all non-AIDS related malignant deaths were from lung cancer and all deaths from lung cancer were in smokers. The life expectancy of smokers was on average 8 years less than that of nonsmokers: the life expectancy of nonsmoking 35-year-old men with CD4^+^ cell count more than 200 cells/μl or viral suppression at least 1 year after starting ART was similar to that of the background population. Excess mortality rates associated with smoking increased substantially with age. At age 65 years, the loss of life years associated with smoking was 7 years, while that associated with HIV-related factors was only 3 years. The finding of a markedly lower risk of death among previous compared with current smokers points towards potential benefits of including smoking cessation interventions in HIV care.

We included individuals from North America and Western Europe who had been on ART for at least 1 year and thus believe that results can be generalized to HIV-infected populations in resource-replete settings with long-term engagement in care. To estimate the loss of life years associated with HIV, we compared the life expectancy of HIV-infected men with that of the male French general population. The life expectancy in France is rather high, but there are only minor differences between Western European countries [20]. Life expectancies are 2–3 years lower in the United States than France [20], and thus, we may have slightly overestimated the loss of life years associated with HIV. We did not have statistical power to estimate life expectancies among women, and therefore, the results on life expectancies and loss of life years can only be generalized to the male HIV-infected population.

The main limitation of the study is the exclusion of a large proportion of participants due to lack of data on smoking status. The distribution of smokers and nonsmokers in the present study was similar to what has been reported from other HIV-infected populations [12,21–23] arguing against differential ascertainment of smoking status for smokers and nonsmokers. Mortality rates were higher among excluded than included individuals, which is likely to result in underestimation of the absolute risk associated with smoking. However, estimates did not differ importantly in analyses including only individuals from the Swiss HIV Cohort, wherein the proportion of participants with known smoking status was highest (96%) or individuals from the Veteran Aging Cohort, wherein data on smoking status were collected at random (data not shown). There was only limited observation time of individuals aged at least 65 years, which may influence the precision of estimates of life expectancies. In sensitivity analyses assuming 20% higher or 20% lower mortality rates in this age group, we found that the estimates of numbers of life years lost in association with smoking varied by 1–2 years compared with primary analyses.

We estimated the impact of ever smoking at baseline rather than including smoking as a time-updated variable. Few individuals start smoking during middle or old age and many of the adverse effects of smoking only manifest after several years of exposure. The risk of death associated with smoking declines gradually after smoking cessation. In an HIV-negative population, it was found that although the increase in risk of death among previous smokers compared with never smokers declined over time since smoking cessation, an increase in risk persisted for up to 19 years [24]. Median follow-up time in the present study was approximately 4 years, and thus change in smoking status for a presumably very small proportion of the study population during the study period is unlikely to change the estimates significantly.

We did not have data on current versus previous smoking from all participating cohorts. In the subgroup of individuals wherein differentiation was possible, previous smokers had similar mortality to never smokers, which is in agreement with findings from previous studies [12,23]. We were unable to directly assess the impact of smoking cessation on life expectancy, but another study found that the incidence of CVD in HIV patients who stopped smoking during follow-up decreased substantially with increased time since smoking cessation [25]. This shows the importance of implementing smoking cessation programmes in the HIV-infected population. Training programmes for HIV physicians in smoking cessation counselling may increase rates of smoking cessation among HIV-infected individuals [26].

Adverse effects of tobacco are unlikely to be the only cause of the higher mortality among smokers than nonsmokers: it is likely that unmeasured confounders, such as alcohol intake and other lifestyle-related factors, contributed to the reduced life expectancy among smokers. Such factors are likely to explain the substantially greater rates of liver-related mortality in smokers than nonsmokers. CD4^+^ cell counts and the proportion with viral suppression at baseline were similar in smokers and nonsmokers, and thus our data do not indicate that smoking is associated with poor HIV control among individuals without injection drug use. In order to estimate the loss of life years associated with being HIV-infected, we compared life expectancies of the HIV-infected population with the French background population. The HIV-infected population differ from the background population not only regarding immune deficiency, immune activation and exposure to ART but also in relation to lifestyle and socio-economic status. Thus, in the era of effective ART, the risk associated with ‘being HIV-infected’ reflects a complex of several risk factors.

The findings of the present study are in line with results of a recent study examining mortality associated with smoking in the HIV-infected population in Denmark [12]. Estimates of the relative risk of death and the numbers of life years lost in association with smoking were somewhat lower in the present study. This may be explained by the fact that we examined the impact of ever rather than current smoking in the present study. Furthermore, the median time from start of ART to study inclusion was shorter and the study population was younger. The mortality in the HIV-infected population is high during the first 2 years after start of ART because of high rates of AIDS-related deaths among late presenters [14,15].

Due to the increase in life expectancy of HIV-infected individuals and the reduction in life years lost to AIDS since the introduction of combination ART [19,27], the impact of smoking and other lifestyle-associated risk factors among HIV-infected individuals is likely to have increased over time concomitantly with ageing of the HIV-infected population. Studies conducted in the pre and early ART era found no association between smoking and mortality [28–30], although more recent studies have shown that smoking is associated with increased morbidity and mortality [23,25,31,32].

We found no indication of interaction between immune deficiency and smoking. The relative risk of death associated with smoking was similar in individuals with high and low CD4^+^ cell counts at baseline, but due to the higher mortality rate among individuals with low CD4^+^ cell counts, the absolute risk associated with smoking is much higher in this group. Due to the lack of an HIV-negative control group with data on smoking status, we could not test for interactions between smoking and HIV, but no interactions were found in a Danish study [12] and our estimates of risk associated with smoking are similar to what has been found in HIV-negative populations [33].

We conclude that HIV-infected individuals with long-term engagement in care may lose more life years through smoking and associated lifestyle factors than through HIV. Excess mortality associated with smoking increases markedly with age; therefore, increases in smoking-related mortality can be expected as the population ages. Interventions for smoking cessation should be prioritized.

## Acknowledgments

We thank all patients, doctors and study nurses associated with the participating cohort studies.

The Corresponding Author has the right to grant on behalf of all authors and does grant on behalf of all authors, a worldwide license. Publishers and its licensees in perpetuity, in all forms, formats and media (whether known now or created in the future), to publish, reproduce, distribute, display and store the Contribution; translate the Contribution into other languages, create adaptations, reprints, include within collections and create summaries, extracts and/or abstracts of the Contribution; create any other derivative work(s) based on the Contribution; to exploit all subsidiary rights in the Contribution; the inclusion of electronic links from the Contribution to third party material wherever it may be located; and licence any third party to do any or all of the above.

No additional data are available.

All authors contributed to the design of the study. N.O. conceived the idea, S.I. and M.M. were responsible for data management, M.H. did the statistical analysis assisted by M.M. M.H. wrote the first draft of the paper assisted by M.M. and J.A.C.S. All authors contributed to interpreting the results and revising the paper. All authors approved the final manuscript. M.H., M.M., S.I. and J.A.C.S. had full access to all data in the study. M.H. is guarantor for the validity of the paper.

Steering group included Andrew Boulle (IeDEA Southern Africa), Hans-Reinhard Brodt (Frankfurt), Jordi Casabona (PISCIS), Matthias Cavassini (SHCS), Geneviève Chêne (Aquitaine), Dominique Costagliola (FHDH), François Dabis (Aquitaine), Antonella D’Arminio Monforte (ICONA), Julia del Amo (CoRIS-MD), Gerd Fätkenheuer (Koln/Bonn), John Gill (South Alberta Clinic), Jodie Guest (HAVACS), David Hans-Ulrich Haerry (EATG), Robert Hogg (HOMER), Amy Justice (VACS), Amanda Mocroft (EuroSIDA), Niels Obel (Denmark), Heidi Crane (Washington), Fiona Lampe (Royal Free), Peter Reiss (ATHENA), Michael Saag (Alabama), Tim Sterling (Vanderbilt-Meherry), Ramon Teira (VACH), Ard Van Sighem (ATHENA), Matthew Williams (UK-CAB), Robert Zangerle (Austria).

Co-ordinating team included Jonathan Sterne and Margaret May (Principal Investigators), Suzanne Ingle (statistician).

The ART Cohort Collaboration is supported by the UK Medical Research Council (grants G0700820 and MR/J002380/1) and the Department for international Development (DFID). J.A.C.S. is supported by NIHR Senior Investigator Award NF-SI-0611-10168. Sources of funding of individual cohorts include the Agence Nationale de Recherche sur le SIDA et les hépatites virales (ANRS), the Institut National de la Santé et de la Recherche Médicale (INSERM), the French, and Italian Ministries of Health, the Swiss National Science Foundation (grant 33CS30_134277), the Stichting HIV Monitoring, the British Columbia and Alberta Governments, National Institute on Alcohol Abuse and Alcoholism (U10-AA13566, U24-AA020794), the US Department of Veterans Affairs, the Michael Smith Foundation for Health Research, the Canadian Institutes of Health Research, the VHA Office of Research and Development and unrestricted grants from Abbott, Gilead, Tibotec-Upjohn, ViiV Healthcare, MSD, GlaxoSmithKline, Pfizer, Bristol Myers Squibb, Roche and Boehringer-Ingelheim. M.H. and N.O. have received funding from the University of Copenhagen.

### Conflicts of interest

J.A.C.S. has received payment for development of educational presentations from Gilead. G.F. is a board member for BMS, Gilead, Merck and Janssen, has grants pending from AbbVie and has received payment for service on speakers bureaus from AbbVie, BMS, Gilead, Janssen and Merck. A.d’A. is a board member for Bristol-Myers Squibb, Abbvie, Gilead, Janssen and Merck Sharp & Dohme-Chibret, and has grants pending from Merck Sharp & Dohme-Chibret, Janssen and Gilead. M.C. has consulted for Bristol-Myers Squibb, Boehringer-Ingelheim, Gilead, Merck Sharp & Dohme-Chibret and Janssen Cilag, has grants pending from Bristol-Myers Squibb, Gilead and Merck Sharp & Dohme-Chibret, has received payment for service on speakers bureaus from Gilead and travel/meeting expenses from Boehringer-Ingelheim, Bristol-Myers Squibb and Gilead. C.S. has funding from BMS, prepared educational material for ViiV, Janssen, BMS, Gilead and attended an Ad board for Gilead. D.C. has received travel grants, consultancy fees, honoraria and study grants from various pharmaceutical companies including Bristol-Myers-Squibb, Gilead Sciences, Janssen-Cilag, Merck-Sharp & Dohme-Chibret, and ViiV Healthcare. P.R. has served as a scientific advisor to Gilead Sciences, and on data and safety monitoring boards committees for Janssen Pharmaceutica, and through his institution received support from Gilead Sciences and Janssen Pharmaceutica for travel to international scientific conferences, and research support from Gilead Sciences, ViiV Healthcare, Tibotec, Bristol Myers Squibb and Merck. F.D. has served as a scientific advisor to Gilead Sciences and Merck & Co and has received honoraria for speaking engagements at workshops and conferences from Gilead Sciences, Inc. J.G. has served on the scientific advisory boards to BMS, Gilead, Janssen, Merck and Viiv Healthcare. M.H. has received travel grants to international scientific conferences from Bristol-Myers-Squibb and Gilead Sciences and a study grant from Gilead Sciences. N.O. has received research funding from Bristol-Myers Squibb, Merck Sharp & Dohme, GlaxoSmithKline, Abbott, Boehringer Ingelheim and Gilead. The remaining authors have no conflicts of interest to declare.

## Supplementary Material

Supplemental Digital Content

## Figures and Tables

**Table 1:** Characteristics of the study population at baseline (≥1 year after start of antiretroviral therapy), *n* (%).

	Smokers	Nonsmokers	*P*^c^
*n*	10 767	7228	
Observation time (years)	49 688	30 072	
Follow-up time (years)^a^	4.3 (1.9–7.3)	3.7 (1.6–6.1)	
Deaths	393	127	
Year of study inclusion^a^	2003 (2001–2006)	2004 (2002–2007)	<0.01
Males	8689 (80.7)	4143 (57.3)	<0.01
Age at baseline (years)^a^	40 (34–47)	38 (32–46)	<0.01
<35	3046 (28.3)	2581 (35.7)	
35–50	5565 (51.7)	3307 (45.8)	
>50	2156 (20.0)	1340 (18.5)	
Route of HIV infection			
MSM	4830 (44.9)	2152 (29.8)	<0.01
Heterosexual male	2746 (25.5)	1476 (20.4)	
Heterosexual female	1906 (17.7)	2852 (39.5)	
Other	1285 (11.9)	748 (10.4)	
AIDS at baseline	2539 (23.6)	1666 (23.1)	0.66
CD4^+^ cell count (cells/μl)^a^^,^^b^	426 (272–614)	390 (254–540)	<0.01
<200	1451 (13.5)	1032 (14.3)	
200–349	2279 (21.2)	1760 (24.4)	
350–500	2371 (22.0)	1831 (25.3)	
>500	3861 (35.9)	2039 (28.2)	
Viral load <400 copies/ml	7759 (72.1)	4952 (68.5)	<0.01
Duration of ART at baseline (years)			
1	7449 (69.2)	6075 (84.1)	<0.01
1–2	764 (7.1)	276 (3.8)	
2–5	1729 (16.0)	540 (7.5)	
>5	825 (7.7)	337 (4.7)	

^a^Median (interquartile range).

^b^At baseline, missing data: CD4^+^ cell count: 1371 cells/μl (7.6); viral load: 1486 copies/ml (8.3).

^c^Mann–Whitney rank sum test for continues data and chi-square test for categorical variables.

**Table 2:** Mortality rates and mortality rate ratios comparing smokers with nonsmokers.

	Observation time (years)	Deaths	MR/1000 person-years (95% CI)		MRR^a^ (95% CI) Smokers versus Nonsmokers
			Smokers	Nonsmokers	
All	79 760	520	7.9 (7.2–8.7)	4.2 (3.5–5.0)	1.94 (1.56–2.41)
Males	58 042	451	8.8 (7.9–9.7)	5.6 (4.6–6.8)	1.84 (1.45–2.34)
Females	21 718	69	4.4 (3.2–5.9)	2.2 (1.5–3.3)	2.41 (1.43–4.08)
Study period of follow-up					
1996–2004	29 728	155	6.2 (5.2–7.4)	3.3 (2.3–4.6)	2.06 (1.36–3.12)
2005–2006	21 879	152	8.2 (6.8–9.9)	4.8 (3.5–6.6)	1.75 (1.18–2.61)
2007–2009	28 153	213	9.8 (8.4–11.5)	4.6 (3.6–6.0)	2.00 (1.43–2.81)
Route of HIV transmission					
MSM	32 272	162	5.6 (4.7–6.6)	3.6 (2.6–5.1)	2.10 (1.39–3.19)
Heterosexual males	18 817	150	9.7 (8.1–11.6)	4.7 (3.3–6.7)	2.19 (1.44–3.35)
Heterosexual females	20 018	57	4.3 (3.1–5.8)	1.7 (1.1–2.7)	3.07 (1.68–5.61)
Other/unknown	5 894	59	19.8 (16.4–23.9)	13.6 (10.1–18.2)	1.31 (0.89–1.92)
Age					
<35 years	26 456	74	3.1 (2.4–4.2)	2.3 (1.6–3.4)	1.71 (0.85–3.42)
35–50 years	38 840	226	7.0 (6.0–8.1)	3.8 (2.9–4.9)	1.77 (1.27–2.48)
> 50 years	14 465	220	18.7 (16.1–21.7)	9.3 (7.0–12.3)	2.14 (1.55–2.95)
AIDS or CD4^+^ cell count <200 cells/μl^b^	24 963	276	13.6 (11.9–15.6)	7.1 (5.6–9.0)	1.91 (1.42–2.59)
No AIDS and CD4^+^ cell count ≥200 cells/μl^b^	54 797	244	5.4 (4.7–6.2)	2.9 (2.2–3.7)	1.90 (1.38–2.61)
Viral load ≤400 copies/ml^b^	51 611	296	6.9 (6.0–7.8)	3.7 (2.9–4.7)	2.01 (1.51–2.66)
Viral load >400 copies/ml^b^	22 598	168	9.4 (7.9–11.1)	4.6 (3.4–6.3)	1.94 (1.35–2.79)
Viral load missing^b^	5 551	56	12.5 (9.2–17.0)	6.6 (4.0–10.9)	0.71 (0.19–2.60)

MR, mortality rate; MRR, mortality rate ratio.

^a^Adjusted for sex, age (time-updated), CD4^+^ cell count at baseline, route of HIV transmission, AIDS at ART initiation, years on ART (time-updated) and calendar year of ART.

^b^At baseline.

**Table 3:** Cause-specific adjusted mortality rate ratios by smoking status.

	All cohorts (79 760 person-years follow-up)		Cohorts with information on current, previous and never-smokers (32 983 person-years follow-up)		
	Deaths	MRR (95% CI) Smoker versus nonsmoker	Deaths	MRR (95% CI) Current versus never smoker	MRR (95% CI) Previous versus never smoker
AIDS-related deaths	152	1.37 (0.93–2.02)	99	1.21 (0.73–2.01)	0.54 (0.28–1.03)
Non-AIDS related deaths	276	2.61 (1.88–3.61)	153	2.45 (1.49–4.03)	1.40 (0.81–2.42)
Non-AIDS malignancies	94	3.13 (1.80–5.45)	43	2.42 (1.03–5.68)	0.94 (0.34–2.64)
Cardiovascular disease	39	6.28 (2.19–18.0)	27	8.82 (1.15–67.8)	4.55 (0.55–37.6)
Non-AIDS infections	26	2.38 (0.88–2.46)	10	3.98 (0.47–15.8)	1.38 (0.12–15.8)
Liver disease	19	8.70 (1.14–66.6)	14	3.44 (0.42–28.4)	1.46 (0.15–14.4)
Other	66	1.42 (0.81–2.49)	29	1.08 (0.40–2.93)	0.66 (0.22–1.98)
Non-AIDS, not classified	32	2.56 (0.96–6.83)	30	1.90 (0.61–5.96)	2.34 (0.73–7.43)
Accident/violence/suicide/substance abuse	36	2.30 (0.92–5.77)	24	2.14 (0.60–7.60)	0.38 (0.06–2.36)
Unknown	56	1.38 (0.78–2.46)	25	0.93 (0.30–2.87)	1.27 (0.41–3.92)

MRRs adjusted for sex, age (time-updated), route of transmission, CD4^+^ cell count at baseline, AIDS at ART initiation, years on ART (time-updated) and calendar year of ART. MRR, mortality rate ratio.

**Table 4:** Life expectancies and potential life years lost among HIV-infected men in association with smoking and HIV-related factors [years (95% confidence interval)].

	Life expectancy				Life years lost	
	Nonsmokers	Smokers	All	All, adjusted^a^	Smoking	HIV-related factors^b^
Age 35 years	41.4 (40.4–42.4)	33.5 (32.9–34.1)	35.3 (34.7–35.9)	38.6 (37.6–39.6)	7.9 (7.1–8.7)	5.9 (4.9–6.9)
Age 65 years	16.1 (15.1–17.1)	9.5 (8.9–10.1)	10.9 (10.3–11.5)	13.7 (12.9–14.5)	6.6 (6.0–7.2)	2.9 (2.1–3.7)
Age 35, CD4^+^ cell count ≥200 cells/μl	43.4 (42.3–44.3)	37.8 (37.0–38.6)	39.3 (38.7–39.9)	41.4 (40.4–42.4)	5.6 (4.8–6.4)	3.0 (2.0–4.0)
Age 35, VL <400 copies/ml	43.5 (41.7–45.3)	35.0 (34.2–35.8)	36.3 (35.7–36.9)	40.4 (38.8–42.0)	8.5 (6.9–10.1)	4.0 (2.4–5.6)

VL, viral load.

^a^If smoking frequency equal to the French male background population (36% smokers).

^b^Life expectancy of the HIV population adjusted for smoking frequency compared with the background population in France (age 35: 44.4; age 65: 16.6).
