# Supplementary material for: Smoking and life expectancy among HIV-infected individuals on antiretroviral therapy in Europe and North America
Source: AIDS. 2015 Jan 7;29(2):221–9. doi: 10.1097/QAD.0000000000000540 (PMC4284008; doi:10.1097/QAD.0000000000000540)
Supplement: Supplemental Digital Content [file aids-29-221-s001.pdf]

**Appendix: Contributing Cohorts**

[French Hospital Database on HIV (ANRS CO4 FHDH)](#_French_Hospital_Database)

[Italian Cohort of Antiretroviral-Naive Patients (ICONA)](#_Italian_Cohort_of)

[Swiss HIV Cohort Study (SHCS)](#_Swiss_HIV_Cohort)

[AIDS Therapy Evaluation project Netherlands (ATHENA)](#_AIDS_Therapy_Evaluation)

[ANRS CO3 Aquitaine Cohort, France](#_ANRS_CO3_Aquitaine)

[Royal Free Hospital Cohort, London UK](#_Royal_Free_Hospital)

[Köln / Bonn Cohort, Germany](#_Köln_/_Bonn)

[Veteran Aging Cohort (VACS), Connecticut, US](#_Veteran_Aging_Cohort)

**Supplementary table 1:** Characteristics of individuals included and those excluded from the study, n(%)

|  | Included | Excluded |
| --- | --- | --- |
|  |  |  |
| n | 17,995 | 24,250 |
| Deaths | 520 (2.9) | 1,881 (7.8) |
|  |  |  |
| Males | 12, 832 (71.3) | 9,813 (66.3) |
|  |  |  |
| Age at start of ART (years)^1^ | 37 (31-45) | 38 (32-46) |
| <35 | 6,954 (38.6) | 8,767 (36.2) |
| 35-50 | 8,497 (47.2) | 11,132 (45.9) |
| >50 | 2,544 (14.1) | 4,351 (17.9) |
|  |  |  |
| Route of HIV infection |  |  |
| MSM | 6,982 (38.8) | 7,282 (30.0) |
| Heterosexual male | 4,222 (23.5) | 3,623 (14.9) |
| Heterosexual female | 4,758 (26.4) | 4,355 (18.0) |
| Other/unknown | 2,033 (11.3) | 8,989 (37.1) |
|  |  |  |
| AIDS at start of ART | 3,767 (20.9) | 5,225 (21.6) |
|  |  |  |
| CD4 count at start of ART(cells/µL)^1^ | 220 (100-340) | 220 (97-353) |
| <200 | 8,014 (44.5) | 10,968 (45.2) |
| 200-350 | 5,719 (31.8) | 7,055 (29.1) |
| 350-500 | 2,479 (13.8) | 3,572 (14.7) |
| >500 | 1,783 (9.9) | 2,654 (10.9) |
|  |  |  |
| Year of ART initiation | 2002 (1999-2005) | 2001 (1999-2004) |
|  |  |  |
|  |  |  |

^1^median (interquartile range)

**Supplementary table 2:** Number of HIV-infected individuals from each of the participating cohorts and the proportion of individuals in the cohort with available data on smoking status.

| Cohort | n (%) |
| --- | --- |
| ATHENA | 2,618 (39) |
| Aquitaine | 1,140 (88) |
| BCCfE | 1,236 (44) |
| FHDH | 9,620 (33) |
| ICONA | 2,865 (89) |
| Royal Free | 484 (35) |
| SHCS | 4,342 (96) |
| VACS | 960 (13) |
| Total | 17,995 (43) |

**Supplementary table 3:** Non-AIDS malignant deaths among smokers and non-smokers

|  | Smokers | |  | Non-smokers | |  | All | |
| --- | --- | --- | --- | --- | --- | --- | --- | --- |
|  | N | MR/10,000 PY (95%CI) |  | N | MR/10,000 PY (95%CI) |  | N | MR/10,000 PY (95%CI) |
| Lung | 34 | 7.1 (5.1-10.0) |  | 0 |  |  | 34 | 4.4 (3.2-6.2) |
| Hepatocellular carcinoma | 4 | 0.8 (0.3-2.2) |  | 1 | 0.3 (0.04-2.4) |  | 5 | 0.6 (0.3-1.6) |
| Pancreas | 4 | 0.8 (0.3-2.2) |  | 1 | 0.3 (0.04-2.4) |  | 5 | 0.6 (0.3-1.6) |
| Head and neck | 3 | 0.6 (0.2-1.9) |  | 1 | 0.3 (0.04-2.4) |  | 4 | 0.5 (0.2-1.4) |
| Stomach | 2 | 0.4 (0.1-1.7) |  | 2 | 0.7 (0.2-2.7) |  | 4 | 0.5 (0.2-1.4) |
| Urinary bladder | 3 | 0.6 (0.2-1.9) |  | 0 |  |  | 3 | 0.4 (0.1-1.2) |
| Anus | 2 | 0.4 (0.1-1.7) |  | 1 | 0.3 (0.04-2.4) |  | 3 | 0.4 (0.1-1.2) |
| Central nervous system | 2 | 0.4 (0.1-1.7) |  | 1 | 0.3 (0.04-2.4) |  | 3 | 0.4 (0.1-1.2) |
| Breast | 2 | 0.4 (0.1-1.7) |  | 1 | 0.3 (0.04-2.4) |  | 3 | 0.4 (0.1-1.2) |
| Haematological | 2 | 0.4 (0.1-1.7) |  | 1 | 0.3 (0.04-2.4) |  | 3 | 0.4 (0.1-1.2) |
| Prostate | 1 | 0.2 (0.01-1.5) |  | 2 | 0.7 (0.2-2.7) |  | 3 | 0.4 (0.1-1.2) |
| Female internal genital | 2 | 0.4 (0.1-1.7) |  | 0 |  |  | 2 | 0.3 (0.01-1.0) |
| Melanoma | 0 |  |  | 2 | 0.7 (0.2-2.7) |  | 2 | 0.3 (0.01-1.0) |
| Soft tissue | 1 | 0.2 (0.01-1.5) |  | 0 |  |  | 1 | 0.1 (0.01-0.9) |
| Colon/rectum | 1 | 0.2 (0.01-1.5) |  | 0 |  |  | 1 | 0.1 (0.01-0.9) |
| Oesophagus | 1 | 0.2 (0.01-1.5) |  | 0 |  |  | 1 | 0.1 (0.01-0.9) |
| Biliary tract | 1 | 0.2 (0.01-1.5) |  | 0 |  |  | 1 | 0.1 (0.01-0.9) |
| Small intestine | 1 | 0.2 (0.01-1.5) |  | 0 |  |  | 1 | 0.1 (0.01-0.9) |
| Hodgkin | 0 |  |  | 1 | 0.3 (0.04-2.4) |  | 1 | 0.1 (0.01-0.9) |
| Bone | 0 |  |  | 1 | 0.3 (0.04-2.4) |  | 1 | 0.1 (0.01-0.9) |
| Poorly differentiated | 4 | 0.8 (0.3-2.2) |  | 1 | 0.3 (0.04-2.4) |  | 5 | 0.6 (0.3-1.6) |
| Unknown | 6 | 1.3 (0.6-2.8) |  | 2 | 0.7 (0.2-2.7) |  | 8 | 1.0 (0.5-2.0) |
|  |  |  |  |  |  |  |  |  |
| Total | 76 | 15.9 (12.7-19.9) |  | 18 | 6.1 (3.9-9.8) |  | 94 | 12.2 (10.0-14.9) |

MR: mortality rate, PY: person-years

**Supplementary table 4:** Life expectancies and potential life years lost among HIV infected men in association with smoking and HIV-related factors (years (95% CI))

|  | **Model 1** | | | | **Model 2** | | | |
| --- | --- | --- | --- | --- | --- | --- | --- | --- |
|  | Life expectancy  (years (95%CI)) | | Life years lost (95%CI) | | Life expectancy (years(95%CI)) | | Life years lost (95%CI) | |
|  | Non-smokers | Smokers | Smoking | HIV related factors | Non-smokers | Smokers | Smoking | HIV related factors |
|  |  |  |  |  |  |  |  |  |
| Age 35 years | 44.7 (43.5-45.9) | 35.2 (34.6-35.8) | 9.5 (8.7-10.3) | 3.1 (2.1-4.1) | 39.1 (38.1-40.1) | 32.3 (31.7-32.9) | 6.8 (6.0-7.6) | 7.7 (6.7-8.7) |
| Age 65 years | 20.2 (19.2-21.2) | 11.8 (11.2-12.4) | 8.4 (7.8-9.0) | 0.0 (-0.8-0.8) | 13.4 (12.8-14.0) | 7.9 (7.5-8.3) | 5.5 (5.3-5.7) | 5.2 (4.6-5.8) |
| Age 35, CD4 ≥200 | 47.1 (45.9-48.3) | 40.4 (39.6-41.2) | 6.7 (5.9-7.5) | 0.0 (-1.0-1.0) | 40.9 (39.9-41.9) | 36.1 (35.5-36.7) | 4.8 (4.2-5.4) | 5.2 (4.4-6.0) |
| Age 35, VL<400 | 47.3 (45.7-48.9) | 36.9 (36.1-37.7) | 10.4 (9.0-11.8) | 0.8 (-0.6-2.2) | 41.0 (39.8-41.9) | 33.7 (33.1-34.3) | 7.3 (6.5-8.1) | 6.0 (5.0-7.0) |

In these sensitivity analyses mortality rates in individuals aged ≥65 years are assumed to be 20% lower (model 1) and 20% higher (model 2) than in the primary analysis.

*If smoking frequency equal to the French male background population (36% smokers)

^#^Compared with the life expectancy of the background male population in France (age 35: 44.4; age 65: 16.6).
